# Comparative Analysis of Retinal Structural and Angiographic Parameters in Diabetic Patients, Alzheimer’s Disease Patients, and Healthy Controls: An OCT-Based Study

**DOI:** 10.22336/rjo.2024.26

**Published:** 2024

**Authors:** Paul-Gabriel Borodi, Mark Slevin, Andrei Hopulele-Petri, Anca Crainic, Pușa Pop, Iulia-Maria Gavriș, Maria-Monica Gavriș

**Affiliations:** * IOSUD Doctoral School, “George Emil Palade” University of Medicine, Pharmacy, Science, and Technology of Târgu Mureş, Târgu Mureş, Romania; ** “Dr. Constantin Papilian” Military Emergency Hospital Cluj-Napoca, Cluj-Napoca, Romania

**Keywords:** Alzheimer’s disease, diabetes, ocular computer tomography, angiography

## Abstract

**Objective:** This study aimed to investigate the potential connections between Alzheimer’s Disease (AD) and diabetes.

**Methods:** This is a cross-sectional study in which AD and diabetes patients sent by the Psychiatry and Diabetes Departments for ophthalmological screening were observed for inclusion/exclusion criteria. Patients were divided into two comparison groups. The first group (n=3) consisted of the age-matched normal and diabetic patient of the stage 3 AD disease participant. The second group (n=3) was for the stage 5 AD patient with diabetes and normal age-matched controls. Each patient underwent a full ophthalmological examination and SS-OCT (Swept Source-Ocular Computer Tomography) for retinal evaluation.

**Results:** A total of 6 patients (12 eyes) were obtained, three men and three women. In the early AD group, the patient with diabetes showed lower macular thickness compared to other groups. In the nasal-inferior (NI) and temporal-superior (TS) ganglion cell layer (GCL), the AD patient showed statistically significant lower values compared to the other patients. In the moderately severe AD group, we found that the AD patient had lower retinal nerve fiber layer (RNFL) thickness on the temporal side compared to the rest of the patients and both the AD patient and diabetes patient showed lower RNFL thickness in the nasal-superior (NS) quadrant. Also, the foveal avascular zone (FAZ) area was statistically significantly lower for both the diabetes and AD patients compared to the healthy control.

**Conclusions:** In conclusion, distinct retinal findings associated with AD and diabetes in young and elderly patients were revealed in our study. The clinical implications and potential interplay between these conditions need to be elucidated by further research.

**Abbreviations:** AD = Alzheimer’s Disease, SS-OCT = Swept Source – Ocular Computer Tomography, GCL = Ganglion cell layer, RNFL = Retinal nerve fiber layer, FAZ = foveal avascular zone

## Introduction

The retina, an integral part of the central nervous system originating as a developmental extension of the brain, has been extensively documented to undergo significant alterations in individuals with Alzheimer’s disease (AD). Serving as an early site for the detection of disease-specific indicators, such as amyloid β-protein plaques, the retinas of AD patients exhibit notable degeneration of ganglion cells, thinning of the retinal nerve fiber layer, and a decline in axonal projections within the optic nerve, among other aberrations. The abnormalities underscore the complex connection between AD and structural changes within the retinal tissue [**1**].

While the observable alterations in the retinal vasculature in diabetes have commonly led to the perception of retinopathy as predominantly a microvascular disorder, it is important to acknowledge that diabetes can inflict harm beyond the vascular system. Non-vascular cells within the retina are susceptible to damage, causing disruptions in functionality and leading to the loss of various cell types, including ganglion cells, horizontal cells, amacrine cells, and photoreceptors. Consequently, diabetic retinopathy encompasses vascular and neural changes within the retina [**2**,**3**].

AD is a prevalent form of dementia, causing persistent memory loss and caregiving challenges. Early detection remains a significant hurdle, relying on modalities like Magnetic Resonance Imaging (MRI), Positron Emission Tomography (PET), and Single Photon Emission Computed Tomography (SPECT) to study brain changes. However, studies on the retina reveal variations in retinal layers, suggesting the retina as a potential biomarker. Optical Coherence Tomography (OCT) represents a pivotal tool for faster and non-invasive detection, offering detailed insights into retinal structural changes [**4**,**5**].

The objective of this study was to investigate the potential connections between AD and diabetes, specifically assessing whether patients with diabetes are at a higher risk of developing AD compared to the normal population. This exploration was based on the analysis of OCT biomarkers and OCT angiography.

## Methods

This cross-sectional study was conducted at “Dr. Constantin Papilian” Military Emergency Hospital Cluj-Napoca between October 2023 and February 2024. AD and diabetes patients sent by the Psychiatry and Diabetes Departments for ophthalmological screening were observed for inclusion/exclusion criteria. Patients with clinical criteria for AD and subjects without clinical criteria for dementia and MMSE scores greater than 26 and no diabetes were included as control patients. Also, we selected patients with Type II Diabetes Mellitus and no clinical criteria for dementia with an MMSE score greater than 26.

The inclusion criteria were AD patients with normal intraocular pressure, and the ability to undergo a proper OCT exam. Exclusion criteria were patients with glaucoma or intraocular pressure over 21, high refractive errors, active intraocular inflammation, and significant media opacities that precluded fundus imaging. Other important known pathologies, such as neurodegenerative diseases, stroke, or uncertain or indeterminate diagnosis were excluded.

The local ethical committee approved the study protocol, informed consent was obtained from the patients, and all the procedures were performed per the revised form of the Declaration of Helsinki (2008).

**Fig. 1:**
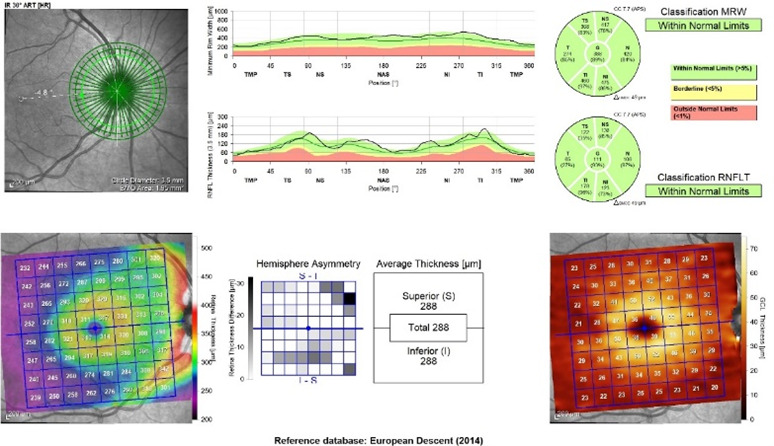
Thickness of retinal nerve fiber layers, retinal thickness, and ganglion cell layer obtained by “Glaucoma premium edition” program

**Fig. 2:**
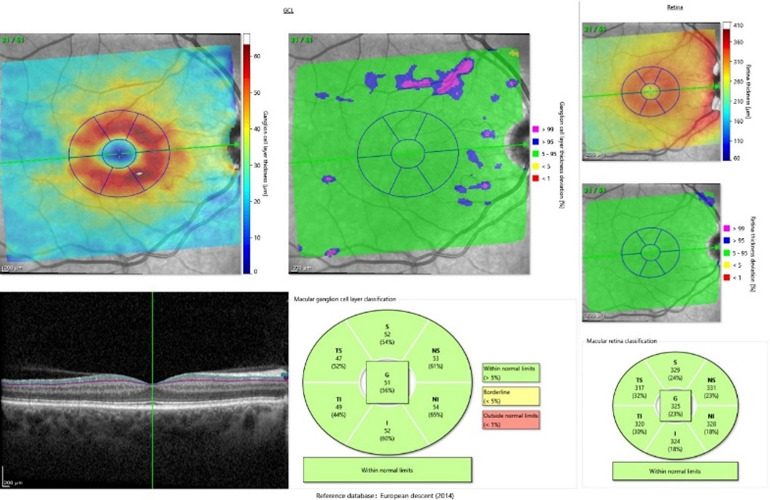
Macular parameters: ganglion cell layer thickness

**Fig. 3:**
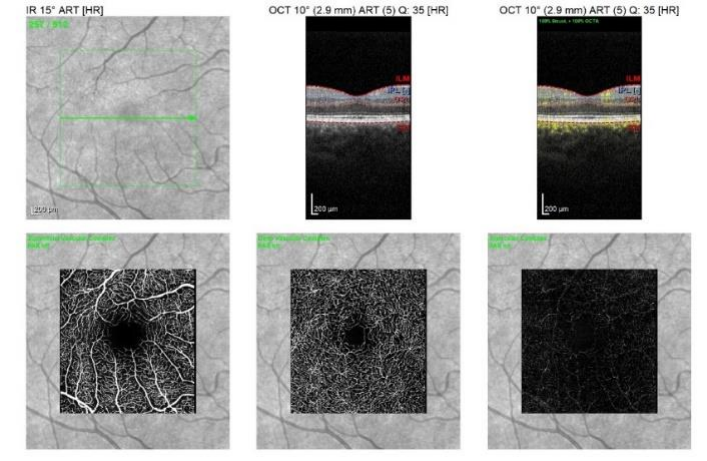
OCT angiography: en face OCT, superficial vascular complex, deep vascular complex, and avascular complex

Each patient underwent a full ophthalmological examination (refraction, visual acuity, intraocular pressure, and biomicroscopy). SS-OCT (Spectralis Heidelberg Engineering, Germany) was used for both eyes of each patient and performed in the same visit for peripapillary retinal nerve fiber layer (pRNFL), macular parameters such as retinal thickness (RT) and ganglion cell layer (GCL) and OCT angiography (OCT-A). The software allows the mapping of thicknesses for seven peripapillary and macular quadrants (**Fig. 1**,**2**). For OCT-A, 3 x 3 mm angiographic scans consisting of 512 A-scans of the macular region were obtained (**Fig. 3**). Each patient had the foveal avascular zone (FAZ) area manually assessed.

Patients were divided into two comparison groups. The first group consisted of the age-matched normal and diabetic patient of the stage 3 AD disease participant. The second group was of the stage 5 AD patient with diabetes and normal age-matched controls. 

Statistical analysis was performed using IBM SPSS Statistics 20 for Windows, Armonk, NY: IBM Corp, USA. One Way ANOVA was used to compare the three groups of patients and Tukey was used as a post-hoc test for the statistically significant differences. To assess the statistical significance of our findings, we adopted a conventional significance level of p < 0.05.

## Results

A total of 6 patients (12 eyes) were obtained, three men and three women. For the AD group, two individuals were included, one presenting with stage 3, mild cognitive impairment (female, 54 years old, MMSE of 18) and the other with stage 5, moderately severe disease (male, 66 years old, MMSE of 10). Additionally, two age-matched participants diagnosed with diabetes (one female, 56 years old, and one male, 63 years old) and two age-matched healthy controls (one female, 55 years old, and one male, 63 years old) were also enrolled in the study. Age matching was performed to ensure group comparability and minimize the potential confounding effects of age differences. 

After fundus examination, no diabetic retinopathy was observed in diabetes patients, while the control group exhibited a normal fundus. In Alzheimer’s disease patients, early-stage individuals presented small cuticular drusen in the macula, while advanced-stage patients showed pigmentary abnormalities with associated soft drusen.

In the early AD group, the patient with diabetes showed lower macular thickness compared to other groups. Compared to the other patients, the AD patient showed statistically significant lower values in the nasal-inferior (NI) and temporal-superior (TS) GCL. We found no other statistically significant differences between patients.

Compared to the rest of the patients in the moderately severe AD group, we found that the AD patient had lower RNFL thickness on the temporal side. Both the AD patient and the diabetes patient showed lower RNFL thickness in the nasal-superior (NS) quadrant. Also, the FAZ area was statistically significantly lower for diabetes and AD patients compared to the healthy control.

## Discussion

Our paper found that in young patients, the GCL can be lower for patients with AD, especially in the NI and TS quadrants. Retinal thickness was lower for the diabetic patient and the RNFL thickness and FAZ did not differ. In the early AD patient, multiple small drusen were found in the macular area. As for the elder group of patients, the GCL did not seem to differ significantly, however, we found that the AD patient had lower RNFL thickness in the NS part. Moreover, FAZ was higher both for diabetes and AD, compared to the control.

Our study results were in concordance with other studies. While Kirbas et al. found lower RNFL thickness in the superior quadrants as we did, and Güneş et al. found a general lowering of this layer. Kesler et al. found that in mild cognitive impaired (MCI) patients the superior RNFL quadrant is more affected, while for AD patients the thinning is generalized [**6**-**8**]. Cunha et al. found that RNFL, GCL, and macular thickness alterations are more prevalent in AD eyes [**9**]. No significant differences were found in the RNFL and GCL thickness between AD, MCI, and normal controls in the study conducted by Sánchez et al. [**10**].

The literature results regarding the FAZ are conflicting and not yet clear. While some authors found that the FAZ area is higher in preclinical AD patients, others found no statistically significant differences. Our results showed differences in the more advanced stage group and no differences for the early disease. Diabetes is a disease mainly microvascular, so having a larger central macular ischemic area as in AD patient, can be interpreted as an early manifestation [**11**-**13**].

Another important transverse finding in this study consisted of the connection between early age-related macular degeneration (AMD) changes and the subsequent onset of AD. A 2011 study demonstrated that oxidative stress can increase β-secretase and γ-secretase activities, leading to elevated Amyloid Beta (Aβ) production - critical in senile plaques formation observed in both AMD and AD. Common pathogenic pathways suggest shared mechanisms, potentially involving oxidative stress and specific activation pathways [**14**]. Oxidative stress boosts BACE1 protein levels through the PKR-eIF2α pathway, contributing to Aβ production. The association of Drusen and AMD with AD indicates a possible link between retinal changes and neurodegenerative processes [**15**,**16**]. Shared characteristics between AMD and AD, such as aging association, unknown etiology, and senile plaques in both the retina and cerebral grey matter, underscore parallel pathogenesis. Essentially, early AMD changes, marked by oxidative stress and Aβ production, may signal a connection to the later development of Alzheimer’s, necessitating further research to uncover precise underlying mechanisms [**17**].

While the current study has provided insights into the potential links between AD and diabetes that have not been addressed in the literature, it is essential to acknowledge certain limitations that warrant consideration. The limited participant pool, underscoring the need for cautious generalization of findings, might have influenced the study’s applicability. Another limitation arose as we were restricted to evaluating quantitatively vascular density or flow index as in other OCTA studies. Future studies should prioritize investigating individuals with diabetes, a population at higher risk, to find potential retinal associations and risk factors that could contribute to the onset of AD.

## Conclusion

In conclusion, our study revealed distinct retinal findings in young and elderly patients associated with AD and diabetes. These findings underscore the potential utility of retinal imaging in identifying early biomarkers for AD and diabetes, emphasizing the need for further research to elucidate the clinical implications and potential interconnection between these conditions.

**Conflict of interest statement**

The authors state no conflict of interest.

**Informed Consent and Human and Animal Rights Statement**

Informed consent was obtained from all individuals included in this study.

**Authorization for the use of human subjects**

Ethical approval: The research related to human use complies with all the relevant national regulations and institutional policies, as per the tenets of the Helsinki Declaration, and has been approved by the review board of “Dr. Constantin Papilian” Military Emergency Hospital Cluj-Napoca, Romania (A11884/20.10.2023).

**Acknowledgments**

None.

**Sources of Funding**

None.

**Disclosures**

None.

## References

[1] Doustar J, Torbati T, Black KL, Koronyo Y, Koronyo-Hamaoui M (2017). Optical Coherence Tomography in Alzheimer’s Disease and Other Neurodegenerative Diseases. Frontiers in Neurology.

[2] Kern TS, Barber AJ (2008). Retinal ganglion cells in diabetes. The Journal of Physiology.

[3] Gastinger MJ, Singh RS, Barber AJ (2006). Loss of cholinergic and dopaminergic amacrine cells in streptozotocin-diabetic rat and Ins2Akita-diabetic mouse retinas. Investigative Ophthalmol & Visual Science.

[4] Sandeep CS, Sukesh Kumar A, Mahadevan K, Manoj P (2019). Early Prediction of Alzheimer’s Disease Using OCT Imaging Technique. Journal of Alzheimer’s Research and Therapy.

[5] Ohno-Matsu K (2011). Parallel findings in age-related macular degeneration and Alzheimer’s disease. Progress in Retinal and Eye Research.

[6] Kirbas S, Turkyilmaz K, Anlar O, Tufekci A, Durmus M (2013). Retinal nerve fiber layer thickness in patients with Alzheimer disease. Journal of Neuroophthalmology.

[7] Güneş A, Demirci S, Tök L, Tök Ö, Demirci S (2015). Evaluation of retinal nerve fiber layer thickness in Alzheimer disease using spectral-domain optical coherence tomography. Turkish Journal of Medical Sciences.

[8] Kesler A, Vakhapova V, Korczyn AD, Naftaliev E, Neudorfer M (2011). Retinal thickness in patients with mild cognitive impairment and Alzheimer’s disease. Clinical Neurology and Neurosurgery.

[9] Cunha LP, Lopes LC, Costa-Cunha LV, Costa CF, Pires LA, Almeida AL, Monteiro ML (2016). Macular Thickness Measurements with Frequency Domain-OCT for Quantification of Retinal Neural Loss and its Correlation with Cognitive Impairment in Alzheimer’s Disease. PLoS One.

[10] Sánchez D, Castilla-Marti M, Marquié M, Valero S, Moreno-Grau S, Rodríguez-Gómez O, Piferrer A, Martínez G, Martínez J, Rojas I, Hernández I, Abdelnour C, Rosende-Roca M, Vargas L, Mauleón A, Gil S, Alegret M, Ortega G, Espinosa A, Pérez-Cordón A, Sanabria Á, Roberto N, Ciudin A, Simó R, Hernández C, Tárraga L, Boada M, Ruiz A (2020). Evaluation of macular thickness and volume tested by optical coherence tomography as biomarkers for Alzheimer’s disease in a memory clinic. Scientific Reports.

[11] Wang X, Zhao Q, Tao R, Lu H, Xiao Z, Zheng L, Ding D, Ding S, Ma Y, Lu Z, Xiao Y (2021). Decreased Retinal Vascular Density in Alzheimer’s Disease (AD) and Mild Cognitive Impairment (MCI): An Optical Coherence Tomography Angiography (OCTA) Study. Frontieres in Aging Neuroscience.

[12] O’Bryhim BE, Apte RS, Kung N, Coble D, Van Stavern GP (2018). Association of Preclinical Alzheimer Disease With Optical Coherence Tomographic Angiography Findings. JAMA Ophthalmology.

[13] López-Cuenca I, Salobrar-García E, Gil-Salgado I, Sánchez-Puebla L, Elvira-Hurtado L, Fernández-Albarral JA, Ramírez-Toraño F, Barabash A, de Frutos-Lucas J, Salazar JJ, Ramirez JM, Ramires AI, de Hoz R (2022). Characterization of Retinal Drusen in Subjects at High Genetic Risk of Developing Sporadic Alzheimer’s Disease: An Exploratory Analysis. Journal of Personalized Medicine.

[14] Mouton-Liger F, Paquet C, Dumurgier J, Bouras C, Pradier L, Gray F, Hugon J (2012). Oxidative stress increases BACE1 protein levels through activation of the PKR-eIF2α pathway. Biochimica et Biophysica Acta (BBA)-Molecular Basis of Disease.

[15] Mody S, Joshi A (2023). Age-Related Macular Degeneration and Its Association With Neurodegenerative Disorders. Cureus.

[16] Busche MA, Konnerth A (2016). Impairments of Neural Circuit Function in Alzheimer’s Disease. Philosophical Transactions of the Royal Society B: Biological Sciences.

[17] Biscetti L, Luchetti E, Vergaro A, Menduno P, Cagini C, Parnetti L (2017). Associations of Alzheimer’s disease with macular degeneration. Front Biosci (Elite Ed).

